# Mefloquine induces cell death in prostate cancer cells and provides a potential novel treatment strategy *in vivo*

**DOI:** 10.3892/ol.2013.1259

**Published:** 2013-03-15

**Authors:** KUN-HUANG YAN, YUNG-WEI LIN, CHI-HAO HSIAO, YU-CHING WEN, KE-HSUN LIN, CHUNG-CHI LIU, MAO-CHIH HSIEH, CHIH-JUNG YAO, MING-DE YAN, GI-MING LAI, SHUANG-EN CHUANG, LIANG-MING LEE

**Affiliations:** 1 Department of Urology, Wan Fang Hospital, Taipei Medical University, Taipei, Taiwan, R.O.C.; 2 Division of General Surgery, Department of Surgery, Wan Fang Hospital, Taipei Medical University, Taipei, Taiwan, R.O.C.; 3 Cancer Center, Wan Fang Hospital, Department of Internal Medicine, Wan Fang Hospital, Taipei Medical University, Taipei, Taiwan, R.O.C.; 4 Center of Excellence for Cancer Research, Department of Internal Medicine, Wan Fang Hospital, Taipei Medical University, Taipei, Taiwan, R.O.C.; 5 Divisions of Gastroenterology, Department of Internal Medicine, Wan Fang Hospital, Taipei Medical University, Taipei, Taiwan, R.O.C.; 6 Hematology and Medical Oncology, Department of Internal Medicine, Wan Fang Hospital, Taipei Medical University, Taipei, Taiwan, R.O.C.; 7 National Institutes of Cancer Research, National Health Research Institutes, Miaoli, Taipei, Taiwan, R.O.C.; 8 Department of Urology, School of Medicine, College of Medicine, Taipei Medical University, Taipei, Taiwan, R.O.C.

**Keywords:** mefloquine, prostate cancer, reactive oxygen species, glutathione

## Abstract

Mefloquine (MQ) is currently in clinical use as a prophylactic treatment for malaria. Previous studies have shown that MQ induces oxidative stress *in vitro*. The present study investigated the anticancer effects of MQ treatment in PC3 cells. The cell viability was evaluated using sulphorhodamine-B (SRB) staining, while annexin V and propidium iodide (PI) were used as an assay for cell death. Reactive oxygen species (ROS) formation was detected with 2′,7′-dichlorofluorescein-diacetate (DCFH-DA), a sensitive intracellular probe, and the alteration of cellular status was defined by trypan blue staining. The results of the present study indicated that MQ has a high cytotoxicity that causes cell death in PC3 cells. MQ markedly inhibited the PC3 cells through non-apoptotic cell death. MQ also induced significant ROS production. The MQ treatment mediated G1 cell cycle arrest and cyclin D1 accumulation through p21 upregulation in the PC3 cells. Moreover, the use of MQ improved the survival of the treatment group compared with the control group in the experimental mice. The present study indicates that MQ possesses potential therapeutic efficacy for the treatment of prostate cancer (PCa) *in vivo*. These findings provide insights that may aid the further optimization and application of new and existing therapeutic options.

## Introduction

The results of clinical trials (1,2) have suggested that chloroquine (CQ) is a possible adjuvant therapy for glioblastoma. We previously observed that mefloquine (MQ) was more potent than CQ in killing glioblastoma cells *in vitro*(3) and that it may prove more efficacious than CQ as a chemotherapeutic agent in hormone receptor-positive and -negative breast cancer cell lines (4). MQ is a prophylactic antimalarial drug that is also used for malaria chemotherapy. MQ is well-tolerated at prophylactic dosages, making it an optimal choice for the majority of travelers. MQ remains a valuable drug for prophylaxis and the treatment of the majority of patients (5). Previous studies have indicated that males have a superior tolerance to MQ compared with females (6). Krudsood *et al*(7) reported that MQ caused a blood plasma concentration of 5,796 ng/ml (15.35 *μ*M) in a clinical study on *Plasmodium falciparum*-infected adults. Dow *et al*(8) noted that higher blood levels of MQ were reached under therapeutic regimens (2.1–23 *μ*M) rather than in prophylaxis (3.8 mM) (9,10).

PC3 cells are isolated from bones with metastatic carcinoma of the prostate. However, p53-null PC3 cells (11,12) are androgen-independent and proliferate normally in androgen-deprived media. In the present study, the cytotoxic efficacy of the quinoline analog, MQ, was investigated in the most commonly used prostate cancer (PCa) cell line, PC3, to determine whether MQ possesses anticancer effects at physiologically relevant concentrations *in vitro* and potential therapeutic efficacy *in vivo*.

## Materials and methods

### Cell culture

The human androgen-independent PC3 cell line was maintained in Dulbecco’s modified Eagle’s medium (DMEM) and supplemented with 10% fetal bovine serum. PCa cells were continuously cultured in a standard cell culture medium with 2 mM L-glutamine, 100 *μ*g/ml streptomycin and 100 U/ml penicillin, in a humidified atmosphere of 5% CO_2_. The study was approved by the Ethics Committee of Taipei Medical University, Wan Fang Hospital, Cancer Center, Taipei, Taiwan.

### Cell viability assay

The PC3 cells were seeded on 96-well plates, then assayed by sulphorhodamine-B (SRB) staining. The SRB staining process was the same as described previously (13). Absorbance was measured at 570 nm using an ELISA reader.

### Cell cycle analysis

The cell cycle was assayed by propidium iodide (PI) staining, followed by Cytomics FC500 Flow Cytometer CXP analysis. Cell cycle profiles were then determined using the CXP analysis software (Beckman Coulter Inc., Miami, FL, USA).

### Western blot analysis

The MQ-treated cells (or the untreated for control) were harvested for western blot analysis. We treated PC3 cells with 10 mM MQ for 24 h. The indicated intervals are showm on Fig 3B. We analyzed cell lysates with western blotting for GAPDH, p21 and cyclin D1. The data is shown for the indicated intervals. Antibodies against GAPDH, p21 and cyclin D1 were purchased from Santa Cruz Biotechnology Inc. (Santa Cruz, CA, USA).

### Flow cytometric assessment of cell death using annexin V/PI assay

A commercially available annexin V apoptosis detection kit (Beckman Coulter Inc.) and flow cytometry were used to identify the annexin V-binding cells. The stained cells were analyzed using a Cytomics FC500 Flow Cytometer CXP. A count of ∼10,000 events was collected for each sample. The percentage distributions of dead cells were calculated using CXP analysis software (Beckman Coulter Inc.).

### Intracellular reactive oxygen species (ROS) assays

To detect ROS formation, a 5 *μ*M non-fluorescent probe, 2′,7′-dichlorofluorescein-diacetate (DCFH-DA), was used as a sensitive intracellular probe. The oxidation of DCFH by ROS was determined by measuring the mean fluorescence intensity of DCFH with flow cytometry (FC500).

### Mouse experiment

Six-week-old male C57BL/6J mice were subcutaneously implanted with 2×10^6^ PC3 cells on the left flank, above the hind limb, on day 1. The mice were then randomly divided into two groups. The mice (control, n=4; MQ-treatment, n=4) weighed 24.0±0.6 g in the control group and 24.0±0.9 g in the treatment group. On days 32, 36, 39 and 43, the control group was treated with 200 *μ*l phosphate-buffered saline (PBS; 1% DMSO), while the treatment group was treated with 200 *μ*g MQ per 25 g of body weight (1% DMSO in PBS/200 *μ*l/mouse) by intraperitoneal (IP) injection. Body weights were measured on days 32, 39, 43 and 47, and survival was monitored continuously between days 1 and 51. The data of the surviving mice were consequently analyzed (n=4 per group).

### Statistical analysis

Error bars represent the standard error of the mean (SEM) from independent triplicates (n=3). All data are expressed as the mean ± SEM. Sigma Plot 2001 software was used for the statistical analysis. P<0.05 was considered to indicate a statistically significant difference.

## Results

### Effects of MQ on the proliferation of PC3 cells

**Figure 1:**
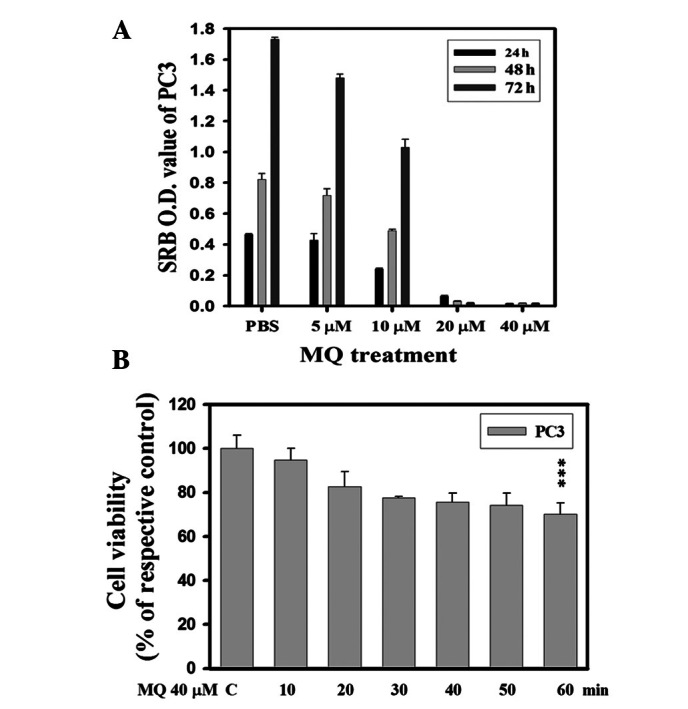
Proliferation inhibition by MQ in PC3 cells *in vitro*. (A) PC3 cells were treated with the indicated concentrations of MQ or PBS (control) for 24, 48 and 72 h, then assayed using SRB staining. (B) PC3 cells were treated with 40 *μ*M MQ for the indicated times. Relative cell viability (% of PBS control) is expressed as the mean ± SEM. Error bars show SEM (n=3). ^*^P<0.05 vs. control; ^**^P<0.01 vs. control.; ^***^P<0.001 vs. control. MQ, mefloquine; PBS, phosphate-buffered saline; SRB, sulphorhodamine-B; O.D., optical density.

The PC3 cells were studied for their sensitivity to MQ using SRB staining *in vitro*. MQ exhibited an IC_50_ of <20 *μ*M (Fig. 1A). The results showed that the IC_50_ value of MQ was around the clinically achievable concentrations for the PC3 cells. The data revealed that 10 *μ*M MQ achieved the IC_50_ at 24 h in one treatment, with no further cytotoxicity at 48 and 72 h exposure. It appeared that the drug was depleted rapidly by the intercellular metabolism. Additionally, 40 *μ*M MQ possessed a high cytotoxicity that caused ∼30% cell death following 60 min of treatment (Fig. 1B).

### Effects of MQ on non-apoptotic cell death in PCa cells

**Figure 2:**
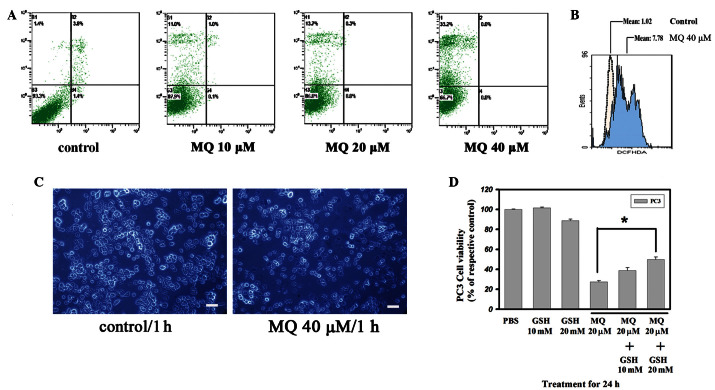
MQ-induced cell death and ROS generation in prostate cancer (PCa) cells. (A) PC3 cells were treated with the indicated concentrations of MQ for 1 h in 6-well plates and the positive staining of cells by annexin V or PI binding was measured with flow cytometry. (B) The PC-3 cells were then treated with MQ (40 *μ*M) for 1 h and the ROS levels were determined with a DCFH-DA dye. (C) The PC3 cells were treated with 40 *μ*M MQ for 1 h in a 96-well plate. The positive trypan blue staining of the cells was then measured and images were obtained with an inverted microscope. Increasing death in PC3 cells was revealed in trypan blue-staining positive cells for MQ-treated cells compared with control cells (Bar=100 *μ*m). Magnification; 4×10. (D) The PC3 cells were pretreated with the the indicated GSH concentrations for 30 min prior to incubation with MQ (20 *μ*M) for 24 h and subsequent monitoring of cell viability. Relative cell viability (% of PBS control) is expressed as the mean ± SEM. Error bars show SEM (n=3, ^*^P<0.05). MQ, mefloquine; ROS, reactive oxygen species; PI, propidium iodide; DCFH-DA, 2′,7′-dichlorofluorescein-diacetate; GSH, glutathione; PBS, phosphate-buffered saline.

The levels of annexin V/PI staining were analyzed following the treatment with MQ for 1 h in the PC3 cells (Fig. 2A). The number of annexin V-stained cells in the 10–40 *μ*M MQ-treated PC3 cells did not increase after 1 h. However, the number of PI stain-positive cells treated with 10 *μ*M MQ increased from 1.4 to 11.0% in the PC3 cells (20 *μ*M MQ, 13.7%). Furthermore, MQ rapidly reduced the population of viable PC3 cells following MQ treatment at 40 *μ*M. The proportion of dead cells among the PC3 cells treated with 40 *μ*M MQ increased to 33.2%. Higher doses of MQ did not increase the number of annexin V-stained cells compared with PI stain-positive cells. Furthermore, numerous trypan blue stain-positive cells appeared in the group treated with 40 *μ*M MQ (Fig. 2C). The data indicated that MQ rapidly caused an increase in the rate of non-apoptotic cell death.

### Effect of MQ on ROS in PC3 cells

Since MQ has a rapid effect on cytotoxicity, the alterations to the levels of ROS were analyzed (Fig. 2B). The data showed that treatment with 40 *μ*M MQ for 1 h caused PC3 intracellular ROS (DCFH) levels to increase significantly to levels 7.6-fold higher than those of the untreated cells. To demonstrate the role of ROS in MQ-induced anticancer effects, cell viability was detected in the PC3 cells pretreated with glutathione (GSH). The MQ-induced anticancer effects were significantly antagonized in the GSH pretreated cells (Fig. 2D), indicating that MQ-mediated cytotoxicity may be quenched by the antioxidant GSH. These data may also suggest that increased ROS generation is essential in MQ-mediated cell death.

### Effect of MQ on cell cycle regulation in PC3 cells

**Figure 3:**
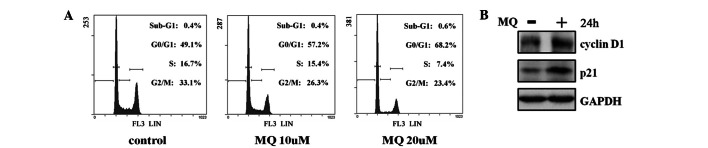
MQ-mediated cell cycle regulation in PC3 cells. (A) The percentage of cells in the G_0_/G_1_, S, G_2_/M and sub-G_1_ phases was determined with flow cytometry. Cells were treated with MQ for the durations indicated and assayed with PI staining. (B) MQ-mediated cell cycle regulation molecules were also assayed. The PC3 cells were incubated in 6-well plates for 24 h, then treated with 10 *μ*M MQ for 24 h. The cell lysates were analyzed with western blotting for p21 and cyclin D1. MQ, mefloquine; PI, propidium iodide.

Using PI staining by flow cytometry, the effect of the MQ treatment on cell cycle regulation was examined (Fig. 3A). The 10 *μ*M MQ treatment for 24 h caused no significant alteration to the proportion of sub-G_1_ PC3 cells, however the MQ treatment significantly induced G_1_ cell-cycle arrest and reduced the proportion of cells in the S and G_2_/M phases. Higher doses of MQ (20 *μ*M) did not increase the number of sub-G_1_ cells in the PC3 cells, but enhanced the G_1_ accumulation from 49.1% (control) to 68.2%. In the western blot analysis (Fig. 3B), 10 *μ*M MQ caused an accumulation of the G_1_ phase marker, the cyclin D1 protein, thus indicating the involvement of p21 upregulation.

### Potential therapeutic efficacy of MQ in an animal model

**Figure 4:**
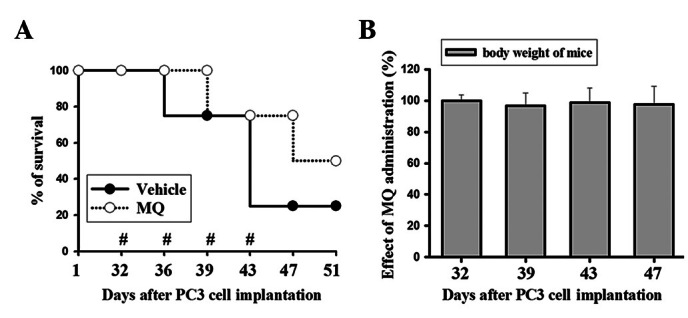
Potential therapeutic efficacy of MQ determined *in vivo*. (A) C57BL/6J mice (6-week-old males; control n=4; MQ-treatment n=4) were subcutaneously implanted with 2×10^6^ PC3 cells, then randomly divided into two groups on day 1. On days 32, 36, 39 and 43, the mice were treated with or without MQ by intraperitoneal (IP) injection. # denotes that the mice were treated with MQ or vehicle via IP on these days. Survival was monitored continually and the data of the surviving mice was analyzed. (B) Effects of IP injection of MQ on the body weight of mice. The body weight of the mice was measured on days 32, 39, 43 and 47. The effects of the administration of MQ on the body weight of the mice and other data are expressed as the mean ± SEM (n=3 or 4 per group). MQ, mefloquine.

The potential therapeutic efficacy of MQ is shown in Fig. 4A. The survival of mice in the MQ group was prolonged compared with those of the vehicle-control group. The MQ-treated group had an increased lifespan and 75% of the mice were still alive at 47 days subsequent to the cancer cell implantation. Moreover, 50% of the mice were still alive at 51 days in the MQ-treated group. By contrast, only 25% of the mice in the control group survived to 47 days post-tumor implantation. MQ treatment improved the survival rate of the mice with tumors. The body weights were measured to evaluate the MQ toxicity caused by the IP injection with 200 *μ*g MQ per 25 g of mouse weight. The results showed no significant alterations in the body weights during the MQ administration period between days 32 and 47 (Fig. 4B). Additionally, as the mice died rapidly after losing control of tumor growth in the control group, the mean tumor size among the survivors was not significantly different between the two groups.

## Discussion

The mechanism of action of MQ was investigated in androgen-independent PCa cells as a model of aggressive PCa in the present study. The PC3 cells were sensitive to the anticancer effects of MQ at ∼10 *μ*M. The results showed that the IC_50_ value of MQ was at clinically achievable concentrations for the PC3 cells. However, treatment with MQ for 24 h induced G_1_ phase cell-cycle arrest in p53-null PC3 cells, although MQ did not induce cell apoptosis. The accumulation of the G_1_ phase marker, the cyclin D1 protein, was consistent with an increased proportion of cells in the G_1_ phase of the cell cycle. The derived results revealed that MQ-mediated G_1_ cell-cycle arrest was p53-independent. By contrast, an increase in p21 was induced in the PC3 cells. The biological functions of p21 regulation have been shown to be emphasized in p53-independent tumor suppressor activities against cancer (14), suggesting that p21 is crucial for MQ-mediated G_1_ cell-cycle arrest in p53-null PC3 cells and that MQ has marked anticancer capability at physiologically achievable concentrations *in vitro*. The present study also investigated the cytotoxicity of MQ in PC3 cells. Cell death rapidly occurred within 1 h of a 10–40 *μ*M MQ exposure, as measured by an annexin V/PI double-staining assay. Consistent with the results of the PI-staining, a significant quantity of trypan blue stain-positive cells was observed in the group treated with 40 *μ*M MQ. Moreover, the ratio of non-apoptotic cell death in the annexin V/PI double-staining assay was consistent with the SRB cell viability data for the 40 *μ*M MQ treatment. The results also revealed that no significant early apoptosis was caused by MQ, even at a concentration of 40 *μ*M.

Evidence suggests that ROS may be used as a therapeutic modality to kill cancer cells. Previous studies have indicated that MQ treatment induces a decreased concentration of GSH that is dependent on the increase in oxidative stress in primary rat cortical neurons (15). ROS generation is an early molecular event that precedes cell death (16). In certain types of necrosis, an increase in the overproduction of ROS mediates cell death (17). A previous study has shown that ROS cause oxidative stress-induced necrotic cell death (18). In the present study, necrotic cell death was accompanied by significant ROS generation, as shown in Fig. 2. This suggested that the MQ treatment mediated the intracellular ROS, allowing the non-apoptotic cell death of the PC3 cells. Pre-treatment of the PC3 cells with GSH abrogated ROS induction by MQ and significantly protected the PC3 cells against MQ-induced anti-cancer effects. These results support the hypothesis that MQ induces ROS generation, which subsequently affects cancer cell proliferation.

Moreover, the present study investigated the potential therapeutic efficacy of MQ in mice (Fig. 4). The results revealed that MQ may contribute to anticancer efficacy by prolonging the survival time of those treated. The MQ-treated group survived longer than the mice of the control group. This suggests a novel strategy that offers a potential new therapy for the treatment of PCa. The results demonstrated that MQ was effective at prolonging the survival time of mice. Although the details remain unclear, the observations of the present study are an initial attempt toward identifying novel treatments for PCa. MQ may be a promising regimen of cancer therapy or tertiary chemoprevention in the future.

Effective and less toxic treatments are the goal of cancer therapy. Since current treatments for advanced PCa are limited, the anticancer effects of MQ provide an attractive target for study. The present results indicate that MQ may be a potential candidate for clinical trials of its cancer therapy applications in the future. Future research may determine whether MQ is able to further synergize with traditional chemotherapy drugs or radiotherapy in the treatment of PCa.

## Abbreviations:

MQ: mefloquine
PCa: prostate cancer
ROS: reactive oxygen species
GSH: glutathione
